# The association of social determinants of health with dental care use and expenditures among older adults in the United States

**DOI:** 10.3389/froh.2026.1870576

**Published:** 2026-10-29

**Authors:** Maryam Alfayyadh, Jay Sureshbhai Patel, Preeti Pushpalata Zanwar, Usha Sambamoorthi

**Affiliations:** 1 Department of Biomedical Sciences, University of North Texas Health, Fort Worth, TX, United States; 2 Department of Oral Health Sciences, Center for Artificial Intelligence, Data Science, and Informatics, Temple University Kornberg School of Dentistry, Philadelphia, PA, United States; 3 NIA Funded Center on Healthy Aging Behaviors & Longitudinal Investigations, University of Chicago, Chicago, IL, United States; 4 College of Pharmacy, University of North Texas Health Science Center, Fort Worth, TX, United States; 5 Department of Pharmacy Practice, Temple University School of Pharmacy, Philadelphia, PA, United States

**Keywords:** dental care utilization, dental expenditures, dental insurance, health services research, medicare policy, older adults, oral health disparities, social determinants of health

## Abstract

**Background:**

Oral health is a critical component of overall health, yet older adults in the United States continue to face persistent disparities in dental care access and utilization, largely shaped by social determinants of health (SDOH).

**Objective:**

This study examined the influence of key SDOH factors on dental care utilization and expenditures among noninstitutionalized adults aged 65 years and older using cross-sectional data from the 2022 Medical Expenditure Panel Survey (MEPS).

**Methods:**

Education, poverty level, marital status, dental insurance coverage, and geographic region were evaluated as primary SDOH variables. Dental care use and expenditures were modeled using a two-part approach in which multivariable logistic regression estimated the likelihood of any dental care utilization and a generalized linear model with log link and gamma distribution assessed total expenditures among users, with marginal effects calculated to estimate combined impacts.

**Results:**

In 2022, 52.5% of older adults reported at least one dental visit, and the average expenditure among users was $1,016. Lower education (adjusted odds ratio [aOR] = 0.25; 95% CI: 0.18, 0.35) and poverty (aOR = 0.51; 95% CI: 0.38, 0.69) were associated with lower dental care use and reduced expenditures (−$161 and −$303, respectively), whereas private dental insurance was associated with higher utilization (aOR = 1.76; 95% CI: 1.42, 2.17) and greater expenditures (+$157).

**Conclusions:**

These findings indicate that structural socioeconomic factors remain strongly associated with dental care access and financial burden among older adults and support the need for policies that integrate oral health into Medicare and broader national strategies to improve access and outcomes.

## Introduction

Oral health is a vital component of overall health and quality of life, as recognized by the World Health Organization, which identifies oral diseases as major noncommunicable conditions affecting general health outcomes (1). A landmark US federal report, published 20 years after the original Surgeon General's Report on oral health, provides robust evidence that oral health is integral to general health and well-being, emphasizing its links to nutrition, speech, social engagement, and chronic disease prevention (2). However, oral health remains one of the most neglected aspects of health care, particularly among older adults in the U.S. and globally. Oral diseases, such as periodontitis, remain one of the most prevalent global health conditions and impose a major burden of disability among people aged 70 and older. Older adults with multimorbidity have complex oral health needs that profoundly affect not only physical health (3) but also communication, social participation, frailty, and overall well-being in later life (4). There is also evidence that preventive periodontal interventions can reduce long-term healthcare costs (5). Preventive and restorative oral health services are essential for preserving function, comfort, and quality of life, yet many individuals, especially older adults, face persistent barriers to obtaining such care. These barriers are rooted not only in clinical or behavioral factors but in the broader social and economic conditions often known as social determinants of health (SDOH). Because oral health disparities among older adults are the cumulative outcomes of social and economic inequities over the life course (6), understanding the role of income, education, and access to care is central to understanding and addressing the disproportionate burden of oral health care utilization. Thus, SDOH is an essential domain for improving oral health because it explicitly links structural, community, interpersonal, and individual factors to oral health care access and outcomes (7).

Older adults in the U.S. are particularly vulnerable to access and affordability constraints. As individuals retire and transition to fixed incomes, they often lose employer-sponsored dental benefits and must rely on out-of-pocket payments or supplemental plans. The policy environment further compounds these challenges, as Medicare, the primary insurer for older adults, does not typically include routine dental coverage. For example, among those with Medicare coverage, approximately 1 in 5 traditional Medicare beneficiaries had a stand-alone dental insurance plan, whereas roughly 62% of those enrolled in Medicare Advantage were in plans offering some dental benefits (8). Even among those with coverage, beneficiaries pay the majority of dental costs out of pocket, reflecting the narrow scope and low reimbursement of available plans (8). A growing body of research links this coverage and cost environment to dental care among older adults. Simulation analyses suggest that extending dental insurance to uninsured older Americans would narrow gaps in dental use and expenditures (8, 9), and studies of demand show that limited dental insurance and high out-of-pocket costs constrain the use of preventive and restorative dental services (10). Even when benefits are available, financial protection is incomplete: seniors enrolled in Medicare Advantage plans with dental benefits continue to report unmet dental need (11). Although these findings are not specific to older adults, they primarily reflect patterns among older adults, who comprise the vast majority (90%) of the Medicare population (12).

Recent studies have underscored how factors such as income, education, and dental insurance play a central role in dental care access and expenditures. Results from the 2021 State of Oral Health Equity in America survey, a nationally representative survey of adults aged 18 years or older on oral health indicated that while lower education and lower income were associated with lower odds of a dental visit in the past 12 months, other SDOH such as food insecurity, housing instability, and transportation barriers were not associated with visiting a dentist in the past 12 months (13). A cross-sectional study of adults ages 18 years or older (*N* = 27,886) from the All of Us research found that no insurance coverage, annual income less than $35,000, and housing instability were all significantly associated with lack of dental care (14). Similarly, Meyerhoefer and colleagues showed that limited dental insurance and high out-of-pocket costs constrain demand for preventive and restorative dental services among older adults (10).

Among older adults aged 65 years or older, one in 10 individuals had untreated caries, indicating a lack of dental care utilization, with a higher prevalence among those with high poverty and less than a high school education (15). Data from the US 2022 National Health Interview Survey suggests that dental care use among U.S. adults aged 65 and older increased steadily with income. Older adults living below the federal poverty level were the least likely to have had a dental visit within the past year, while those in higher-income brackets are about twice as likely to receive dental care. This pattern highlights a clear income-related disparity in oral-health service utilization among older Americans (16). Consistent with these income-related patterns, among low-income adults aged 65 and older, residence in states with more generous Medicaid adult dental benefits is associated with a higher likelihood of a dental visit (17). A mini review of oral health access among aging adults in the U.S. reported that older adults, aged 65 or older, with low income or inadequate insurance, are significantly less likely to receive regular dental visits and more likely to defer care due to cost or coverage barriers (18).

While national surveys provided valuable, nationally representative insights into how SDOH correlate with dental care access among U.S. adults, these studies primarily examine the general adult population and offer limited insight into the unique socioeconomic and insurance-related factors influencing dental care utilization and expenditures among older adults. Furthermore, these surveys do not capture detailed information on dental care costs or out-of-pocket expenditures. The cited Medicare-based analyses (e.g., Simon & Cai (19); Willink et al. (8)) describe the Medicare population broadly rather than adults aged 65 and older specifically, and the national SDOH surveys examine the general adult population. We estimate a two-part model that jointly characterizes the probability of any dental care use and the level of expenditure among users, accommodating the large share of older adults with no dental spending in a given year. To our knowledge, few studies provide nationally representative estimates of both dental care use and expenditures, with simultaneous adjustment for a comprehensive set of social determinants, among adults aged 65 and older.

To address this critical gap, the present study uses the nationally representative Medical Expenditure Panel Survey (MEPS) to examine how key SDOH, such as poverty, education, dental insurance, marital status, and region, affect dental care utilization and expenditures among adults aged 65 years and older in the US.

## Methods

### Study design and data source

This study employed a cross-sectional design using data from the 2022 MEPS. MEPS is a nationally representative dataset that captures detailed information on health status, healthcare utilization, medical expenditures, and insurance coverage among the U.S. civilian noninstitutionalized population. Because this study utilized publicly available, de-identified data, it was exempt from institutional review board approval. MEPS is recognized for its rigorous sample design, stratification, and weighting methodologies (20).

#### Analytical sample

The analytic sample consisted of adults ages 65 years and older (Unweighted *N* = 5,115), representing 57,585,430 older adults, on whom data was collected via in multiple rounds of interviews in 2022 (21).

##### Exclusion criteria

Because lacking any health insurance coverage is itself a dominant barrier to care, we focus on a coverage-eligible (insured) population to isolate the contributions of other social determinants and excluded those without insurance coverage (*N* = 33). We also excluded those who were not fully observed through the calendar year (*N* = 49).

##### Missing data

Missingness was limited for education (*N* = 46; weighted 1.0%), smoking status (*N* = 44; weighted 1.0%), and physical activity (*N* = 52; weighted 1.2%). To avoid changes in the analytic denominator across models, we included missing-indicator categories for these covariates rather than excluding observations with missing values. This approach allowed the study sample size to remain consistent across all analyses.

### Measures

#### Dependent variables

##### Dental care use

Data for annual dental visits were based on dental events reported by household respondents and recorded in the dental visits file provided by the MEPS investigators (22). Adults with dental visits and non-zero expenditures were defined as dental care users.

##### Dental care expenditures

MEPS defines expenditures as payments for dental care services (22). Total expenditures in 2022 for dental care were derived from 10 sources of payments, including self-pay or out-of-pocket, Medicare, Medicaid, Private, Veterans Administration, TRICARE, other federal, other state and local, workers' compensation, and miscellaneous or unknown sources.

### Key explanatory variables: SDOH

SDOH included education level (less than high school, high school graduate, some college, and college graduate), poverty status (poor/near poor: family income <200% of the federal poverty level; middle/high income: ≥200% of the federal poverty level), dental insurance coverage (Medicare or private coverage, Medicare only, and no coverage), marital status (married, widowed/divorced/separated, and never married), and geographic region (Northeast, Midwest, South, and West). The dental coverage information was derived from two survey items: one asking about private standalone dental coverage, and another, for respondents enrolled in Medicare Advantage, asking about dental coverage through their Medicare Advantage plan (AHRQ, 2024) (22).

### Other explanatory variables

These included age (65–74, 75–84, and 85+ years), sex (male, female), race and ethnicity (Non-Hispanic White (NHW), Non-Hispanic Black (NHB), Hispanic, and Other race), polypharmacy defined as taking more than 5 medications during a 180-day period, smoking, body mass index categories, depression, anxiety, and number of chronic conditions (0, 1, 2, and ≥3). The compilation of chronic conditions was based on information obtained from both medical records and household data files. The following chronic conditions were included: arthritis, asthma, coronary heart disease, cardiac arrhythmia, congestive heart failure, cancer, chronic obstructive pulmonary disease, diabetes, dementia, high cholesterol, hypertension, human immunodeficiency virus, hepatitis, osteoporosis, and stroke.

### Statistical analyses

Unadjusted group differences in dental care use were tested with Rao-Scott Chi-square statistics. Descriptive statistics were computed to summarize SDOH and dental care expenditures. For continuous variables, means, 95% confidence intervals (CIs), medians, and interquartile ranges (IQRs) were reported, and group differences were evaluated using survey-weighted tests of mean equality. Unadjusted and adjusted differences in dental care expenditures by SDOH and other characteristics were also tested with two-part models (23) because healthcare expenditure data, including dental expenditures, have distinct characteristics such as a large proportion of zeros (many individuals report no dental care spending in a given time) and highly right-skewed expenditures (a small fraction of individuals incurring very large expenditures). We modeled dental expenditures with a two-part model. The first part was a logistic regression for the probability of any dental expenditure. The second part was a generalized linear model with a gamma distribution and a log link for the level of expenditure among persons with any dental expenditure. Both parts incorporated the complex survey design of MEPS.

To aid interpretation, we calculated average marginal effects on overall (unconditional) expected dental expenditure using postestimation marginal commands. For each categorical predictor, the average marginal effect is the mean difference in predicted dental expenditure (in 2022 US dollars) for a given category relative to its reference category, averaged across the analytic sample. Standard errors were obtained by the delta method and accounted for the survey design. These effects combine the change in the probability of having any dental expenditure (logit model) with the change in conditional spending among users (GLM), providing an interpretable dollar estimate of the total effect (24, 25). Analyses accounted for the complex survey design of MEPS using Stata svy commands (person weight PERWT; strata VARSTR; PSU VARPSU). Estimation was restricted to study population using the subpopulation option (subpop()), which preserves the full MEPS sample for variance estimation and avoids underestimation of standard errors. Statistical significance was defined as *p* < 0.05. Results are presented as odds ratios (ORs) for dental care use and as differences for expenditures, with 95% CI.

## Results

A majority of older adults in the U.S. were female (56.3%) and in the 65–74-year age range (58.6%). Regarding SDOH, 34.1% had completed college, whereas 21.5% had less than a high school diploma. Approximately 25% of participants lived below the federal poverty line, and 41.8% reported having no dental insurance. More than half were married (53.2%), and 38.7% resided in the Southern United States (Table 1).

**Table 1:** Description of older adults (age ≥65 years).

Medical Expenditure Panel Survey, 2022			
Variables	*N*	Wt. N	Wt. %
ALL	5,115	57,585,430	100.0
Sex			
Female	2,894	31,431,555	54.6
Male	2,221	26,153,875	45.4
Age in Years			
65–69 years	1,590	18,202,476	31.6
70–74 years	1,437	15,561,685	27.0
75–79 years	982	10,837,830	18.8
80 or older	1,106	12,983,439	22.5
Race and Ethnicity			
NHW	3,608	42,638,903	74.0
NHB	689	5,618,443	9.8
Hispanic	559	5,311,805	9.2
Other race	259	4,016,278	7.0
Education			
LT HS	730	6,849,710	11.9
HS	1,462	16,588,141	28.8
Some College	1,102	13,891,859	24.1
College	1,775	19,651,141	34.1
Poverty Status			
Poor	799	5,779,254	10.0
Near Poor	1,117	11,084,731	19.2
Middle income	1,337	16,636,215	28.9
High Income	1,862	24,085,231	41.8
Dental Insurance			
Private/Mcare	1,101	13,914,749	24.2
Mcare only	950	10,156,799	17.6
No insurance	3,064	33,513,882	58.2
Marital Status			
Married	2,497	31,298,171	54.4
Wid/Div/Sep	2,262	23,123,023	40.2
Never married	356	3,164,236	5.5
Region			
Northeast	896	10,532,273	18.3
Mid west	1,052	12,313,303	21.4
South	1,934	21,750,611	37.8
West	1,233	12,989,243	22.6
Multimorbidity			
GE 2 chron	4,250	47,091,946	81.8
No mmorb	865	10,493,484	18.2
Anxiety			
Anxiety	515	5,380,610	9.3
No anxiety	4,600	52,204,820	90.7
Depression			
Depression	497	5,170,440	9.0
No Depression	4,618	52,414,990	91.0
Tobacco Use			
Every day	318	3,409,517	5.9
Some days	125	1,253,383	2.2
None	4,628	52,320,016	90.9
Physical Activity			
GE 5 times/Week	2,510	28,640,026	49.7
Other	2,551	28,231,332	49.0
Polypharmacy			
GE 6 classes	1,659	17,495,899	30.4
No polypharmacy	3,456	40,089,531	69.6

Based on 5,115 adults (aged 65 years or older) with health insurance and observed through out the year in 2022. Missing values in the following variables are not presented: education, tobacco use, and physical activity.

Chron, Chronic conditions; HS, High school; GE, Greater than or equal; mcare, Medicare mmorb, Multimorbidity; Wt, Weighted; Wid/div/sep, Widowed or divorced or separated.

### Dental care utilization

Only 52.5% of older adults reported at least one dental visit during 2022 (Table 2). Dental care use varied by SDOH variables. A higher percentage of college-educated older adults (70.3% vs. 26.7%) used dental care than those with less than a high school education. Poverty status showed a similar pattern. A higher percentage of high-income older adults (64.8% vs. 33.5%) used dental care than those living in poverty. Dental insurance coverage was strongly associated with use. A higher percentage of older adults with private dental coverage in addition to Medicare (65.3% vs. 48.0%) used dental care than those without any dental insurance. A higher percentage of married older adults (57.9% vs. 45.8%) used dental care than those who were widowed, divorced, or separated (Table 2).

**Table 2:** Description of older adults (age ≥65 years) by use of dental services.

Medical Expenditure Panel Survey, 2022						
	User		Non-User			
Variables	*N*	Wt. Row %	N	Wt Row %	Chi-square	Prob
ALL	2,629	52.5	2,486	47.5		
Sex					2.053	0.152
Female	1,502	53.6	1,392	46.4		
Male	1,127	51.3	1,094	48.7		
Age in Years					18.687	0.000
65–69 years	829	53.7	761	46.3		
70–74 years	773	55.3	664	44.7		
75–79 years	524	55.6	458	44.4		
80 or older	503	45.1	603	54.9		
Race and Ethnicity				113.18	0.000	
NHW	2,142	58.8	1,466	41.2		
NHB	211	32.1	478	67.9		
Hispanic	170	32.2	389	67.8		
Other race	106	41.5	153	58.5		
Education					325.892	0.000
LT HS	169	26.7	561	73.3		
HS	585	41.0	877	59.0		
Some College	593	55.9	509	44.1		
College	1,274	70.3	501	29.7		
Poverty Status					130.818	0.000
Poor	234	33.5	565	66.5		
Near Poor	440	39.4	677	60.6		
Middle income	673	50.2	664	49.8		
High Income	1,282	64.8	580	35.2		
Dental Insurance				59.259	0.000	
Private/Mcare	729	65.3	372	34.7		
Mcare only	469	50.0	481	50.0		
No insurance	1,431	48.0	1,633	52.0		
Marital Status					48.142	0.000
Married	1,453	57.9	1,044	42.1		
Wid/Div/Sep	1,022	45.8	1,240	54.2		
Never married	154	49.2	202	50.8		
Region					9.638	0.022
Northeast	524	54.7	372	45.3		
Mid-west	591	57.4	461	42.6		
South	854	48.9	1,080	51.1		
West	660	52.3	573	47.7		
Multimorbidity					7.721	0.005
Yes	2,210	53.7	2,040	46.3		
No	419	47.4	446	52.6		
Anxiety					1.819	0.177
Anxiety	265	55.9	250	44.1		
No anxiety	2,364	52.2	2,236	47.8		
Depression					0.383	0.536
Depression	244	50.8	253	49.2		
No Depression	2,385	52.7	2,233	47.3		
Tobacco Use					64.979	0.000
Every day	81	27.0	237	73.0		
some days	30	26.7	95	73.3		
None	2,506	55.0	2,122	45.0		
Physical Activity					25.855	0.000
GE 5 times/Week	1,402	57.0	1,108.0	43.0		
Other	1,210	48.4	1,341.0	51.6		
Polypharmacy					xxx.xxx	0.977
GE 6 classes	836	52.6	823.0	47.4		
No polypharmacy	1,793	52.5	1,663.0	47.5		

Based on 5,115 adults (aged 65 years or older) with health insurance and observed throughout the year in 2022. Missing values in the following variables are not presented: education, tobacco use, and physical activity. Group differences in dental care use were tested using Rao-Scott chi-square statistic.

Chron, Chronic conditions; HS, High school; GE, Greater than or equal; Mcare, Medicare; Wt, Weighted; Wid/div/sep, Widowed or divorced or separated.

### Dental care expenditures

In 2022, the mean total dental expenditure among older adults was $640 (95% CI: $556–$724), with a median expenditure of $111 (IQR: $0–$479) (Table 3). The mean expenditure for those who had at least one dental visit during 2022 was $1,218. Favorable SDOH were associated with higher dental care expenditures. For example, college graduates had higher mean expenditures ($860 vs. $459) than high school graduates. A higher mean expenditure ($789 vs. $569) was observed among those with private or Medicare-private dental coverage compared with those with Medicare-only coverage. Unadjusted two-part models confirmed these findings (Appendix S1) (Table 3).

**Table 3:** Weighted mean, 95% CI, median and IQR of total dental expenditures among older adults (age ≥65 years) medical expenditure panel survey, 2022.

Medical Expenditure Panel Survey, 2022			
Variables	Mean (95% CI)	Median (IQR)	*p*-value
ALL	640 [556,724]	111 [0–479]	
Dental Visits			
Yes	1,218 [1,068,1,368]	449 [239–1,264]	
Sex			
Female	622 [555,688]	120 [0–485]	0.6403
Male (Ref)	662 [501,824]	88 [0–469]	
Age in Years			
65–69 years (Ref)	667 [447,887]	115 [0–463]	
70–74 years	707 [577,837]	140 [0–509]	0.7558
75–79 years	631 [525,738]	148 [0–557]	0.7755
80 or older	529 [440,618]	0 [0–415]	0.2447
Race and Ethnicity			
NHW (Ref)	729 [619,840]	173 [0–558]	
NHB	390 [278,503]	0 [0–221]	<0.001
Hispanic	386 [254,517]	0 [0–185]	<0.001
Other race	378 [246,510]	0 [0–381]	<0.001
Education			
LT HS	519 [−50,1,088]	0 [0–100]	0.251
HS	459 [367,550]	0 [0–298]	<0.001
Some College	630 [535,725]	145 [0–597]	0.0017
College (Ref)	860 [761,958]	287 [0–700]	
Poverty Status			
Poor	348 [234,462]	0 [0–205]	<0.001
Near Poor	395 [310,481]	0 [0–265]	<0.001
Middle income	714 [459,969]	63 [0–488]	0.6713
High Income (Ref)	772 [688,856]	250 [0–608]	
Dental Insurance			
Private/Mcare (Ref)	789 [689,888]	251 [0–698]	
Mcare only	569 [444,694]	0 [0–450]	0.0082
No insurance	600 [466,733]	0 [0–417]	0.0278
Marital Status			
Married (Ref)	648 [580,716]	150 [0–516]	
Wid/Div/Sep	643 [458,829]	0 [0–434]	0.9613
Never married	535 [387,683]	0 [0–431]	0.1672
Region			
Northeast	749 [597,902]	143 [0–600]	0.3106
Mid-west	648 [541,755]	154 [0–539]	0.8849
South	572 [379,764]	0 [0–407]	0.4186
West (Ref)	658 [570,746]	120 [0–477]	
Multimorbidity			
Yes	657 [558,757]	124 [0–500]	0.2709
No (Ref)	563 [433,693]	0 [0–383]	
Anxiety			
Anxiety	684 [503,865]	157 [0–570]	0.6403
No anxiety (Ref)	636 [545,726]	105 [0–468]	
Depression			
Depression	539 [421,657]	74 [0–490]	0.1384
No Depression (Ref)	650 [559,741]	114 [0–475]	
Tobacco Use			
Every day	383 [113,654]	0 [0–105]	0.0577
Some days	306 [104,509]	0 [0–203]	0.0019
	None (Ref)	661 [572,750]	139 [0–504]
Physical Activity			
GE 5 times/Week (Ref)	620 [547,692]	149 [0–498]	
Other	653 [506,800]	0 [0–460]	0.6832
Polypharmacy			
GE 6 classes	749 [514,983]	119 [0–523]	0.2093
No polypharmacy (Ref)	593 [527,658]	109 [0–454]	

Based on 5,115 adults (aged 65 years or older) with health insurance and observed throughout the year in 2022. Missing values in the following variables are not presented: education, tobacco use, and physical activity. Group differences in weighted mean total dental care and out-of-pocket expenditures were tested using t statistics.

Chron, Chronic conditions; HS, High school; GE, Greater than or equal; mcare, Medicare; Wt, Weighted; Wid/div/sep, Widowed or divorced or separate.

#### Multivariable Two-part model of dental care use and expenditures

From the adjusted logistic regression (Part 1), Older adults with lower educational levels were less likely to use dental care (less than high school, adjusted odds ratio (aOR) = 0.25; 95% CI = 0.18, 0.35) compared to those with a college education (Table 4). Poverty (poor and low income) was associated with lower odds of using dental care compared to the group with incomes equal to or above 400% federal poverty line. The odds ratios ranged from 0.51 (95%CI = 0.38, 0.69) for the poor group and 0.61 (95% CI = 0.48, 0.79) for the low-income group. Individuals with private dental insurance were 1.76 times as likely to use dental services compared to those without insurance (aOR = 1.76, 95% CI = 1.42, 2.17). No significant differences were observed for marital status and region categories (Table 4).

**Table 4:** Adjusted Two-part model of dental care use and expenditures among older adults (age ≥65 years) logistic regression on dental care use and generalized linear model (GLM) with gamma distribution and Log link.

Medical Expenditure Panel Survey, 2022								
	Logistic Regression			GLM		Marginal Effects		
	aOR	95% CI	*p*-value)	Beta	SE	Excess $	95% CI	*P*-value
Sex (Ref = Male)								
Female	1.33	[1.15, 1.53]	<0.001	−0.05	0.07	39.18	[−57.36,135.71]	0.4250
Age in Years (Ref = 65–69 years)								
70–74 years	1.10	[0.87, 1.39]	0.4070	0.12	0.11	106.89	[−53.97,267.74]	0.1920
75–79 years	1.07	[0.83, 1.39]	0.5990	−0.01	0.12	7.60	[−152.57,167.77]	0.9260
80, Older	0.78	[0.60, 1.00]	0.0470	−0.03	0.13	−76.73	[−238.62,85.17]	0.3520
Race and Ethnicity (Ref = NHW)								
NHB	0.37	[0.27, 0.50]	<0.0001	0.03	0.13	−251.15	[−419.41,−82.89]	0.0040
Hispanic	0.50	[00.37,0.68]	<0.0001	−0.12	0.18	−241.40	[−448.27,−34.52]	0.0220
Other	0.52	[0.34, 0.79]	0.0020	−0.36	0.16	−338.40	[−504.93,−171.86]	<0.0001
Education (Ref = College)								
LT HS	0.25	[0.18,0.35]	<0.0001	0.37	0.37	−161.62	[−665.13, 341.90]	0.5280
HS	0.34	[0.28, 0.43]	<0.0001	−0.07	0.11	−318.25	[−438.82, −197.68]	<0.001
Some College	0.60	[0.48,0.75]	<0.0001	−0.07	0.09	−174.30	[−305.90, −42.69]	0.0100
Poverty Status (Ref = High Income)								
Poor	0.51	[0.38, 0.69]	<0.0001	−0.25	0.16	−303.95	[−479.23, −128.68]	0.
Low	0.61	[0.48, 0.79]	<0.0001	−0.26	0.10	−270.18	[−408.65, −131.71]	<0.001
Middle	0.79	[0.62, 1.01]	0.0600	0.04	0.10	−34.98	[−186.40, 116.44]	0.6500
Dental Insurance (Ref = None)								
Private	1.76	[1.42, 2.17]	<0.0001	0.02	0.09	157.26	[36.69, 2 77.83]	0.0110
Mcare only	1.33	[1.05, 1.68]	0.0160	0.00	0.12	74.65	[−78.94, 228.23]	0.3390
Marital Status (Ref = Married)								
Wid/Div/Sep	0.88	[0 0.73, 1.05]	0.1620	0.19	0.09	−88.21	[−47.32, 223.74]	0.2010
Single	1.05	[0.77, 1.44]	0.7400	−0.03	0.14	−6.90	[−182.55, 168.75]	0.9380
Region (Ref = Northeast)								
Midwest	1.01	[0.74, 1.39]	0.9330	−0.20	0.10	−131.97	[−289.51, 25.56]	0.1000
South	0.82	[0.61, 1.09]	0.1680	−0.19	0.10	−175.35	[−317.44, −33.26]	0.0160
West	0.91	[0.67, 1.23]	0.5220	−0.01	0.11	−35.03	[−186.98, 116.93]	0.6500
Multimorbidity (Ref = No)								
Yes	1.60	[1.29, 1.99]	< 0.0001	−0.08	0.11	71.58	[−72.01, 215.17]	0.3270
Depression (Ref = No)								
Yes	0.79	[0.60, 1.06]	0.1130	−0.21	0.13	−170.85	[−309.61, −32.08]	0.0160
Anxiety (Ref = No)								
Yes	1.21	[0.92, 1.59]	0.1730	0.11	0.17	124.80	[−129.17, 378.77]	0.3340
Physical Activity (Ref = Other)								
>5 times/wk	0.86	[0.73, 1.00]	0.0540	0.14	0.07	52.09	[−45.55, 149.72]	0.2940
Tobacco Use (Ref = No)								
Every Day	0.38	[0.26, 0.57]	<0.0001	0.21	0.33	−140.45	[−512.90, 232.00]	0.4580
Some days	0.32	[0.17, 0.61]	0.0010	−0.14	0.25	−326.04	[−517.30, −34.78]	0.0010
Polypharmacy (Ref = No Polypharmacy)								
Yes	1.13	[0.93, 1.37]	0.2230	0.16	0.09	139.41	[10.69, 268.12]	0.0340

Based on 5,115 older adults (aged 65 years or older) with health insurance and observed throughout the year in 2022. Missing values in the following variables are not presented: education, tobacco use, and physical activity.

HS, High school; GE, Greater than or equal; Mcare, Medicare; NHB, Non-Hispanic Black, Wid/div/sep, Widowed or divorced or separated; wk, Week.

### Marginal effects

Using marginal effects reflects both their lower likelihood of having a dental visit and their lower level of spending among those who received care. We found that, compared with college graduates, older adults with a high school education spent $318 less per year on dental care. Similarly, those with some college had lower expenditures (−$174; 95% CI = −305.90—−42.69).

Older adults with Medicare-only coverage spent $331 less annually than those with private dental insurance, after combining differences in both the probability of use and conditional expenditures. Compared with older adults earning 400% above the federal poverty line, those living in poverty spent an estimated $304 less per year on dental care (95% CI: $129–$479 less), after combining differences in both the probability of any use and conditional expenditures. Low-income older adults spent about $270 less annually than their high-income counterparts (95% CI: $132–$409 less). Dental spending among middle-income older adults did not differ significantly from that of high-income adults (−$35; 95% CI: $116 less to $186 more). Older adults with private dental insurance spent an estimated $157 more per year on dental care than those without dental coverage (95% CI: $37 to $278; *p* = 0.011). In contrast, there were no significant differences between older adults with Medicare-only coverage and those with no dental coverage.

## Discussion

This cross-sectional analysis of MEPS 2022 data revealed that only 1 in 2 adults age 65 years and older in the U.S. had a dental care visit in 2022, somewhat lower than NHIS estimates (63.7%). The difference likely reflects our definition of a dental care user as having both a dental visit and non-zero expenditure, which excludes visits without recorded expenditures, together with known differences between MEPS and NHIS in how dental visits are captured. Because we also restricted the sample to those with general health coverage, which would tend to raise utilization, the lower estimate most plausibly reflects this conservative, expenditure-based definition rather than lower underlying use.

The two-part model revealed that SDOH were associated with dental care at two critical stages. The first part revealed a lower likelihood of older adults receiving any dental care for lower levels of education and poverty, indicating that these barriers often prevent individuals from even entering the care system, and similar to what has been previously reported on these gradients for dental care use (26). Our results are in line with other studies showing that education and poverty were associated with underuse of preventive dental services among older adults (27). Older adults with dental insurance were more likely to have a dental visit, consistent with coverage reducing the out-of-pocket cost and financial uncertainty of seeking care (10). These findings indicate that socioeconomic resources and coverage shape whether older adults enter dental care, and that dental insurance, unlike structural socioeconomic disadvantage, remains a modifiable point of intervention (28).

In the second stage, SDOH were associated with the level of spending among those who received care. There are several plausible interpretations. Lower education and income were associated with lower dental expenditures, suggesting that socioeconomic disadvantage may also reduce the intensity or comprehensiveness of care among users. However, this pattern should be interpreted cautiously: lower spending may reflect fewer services, lower-cost procedures (for example, extractions rather than restorative or prosthodontic treatment), or a higher prevalence of edentulism in disadvantaged groups, rather than underuse alone. In contrast, private dental insurance was associated with higher total expenditures. Because we measured total payments from all sources, higher spending among the insured most plausibly reflects greater receipt of care, including restorative or elective services that uninsured older adults may forgo rather than a greater financial burden; the out-of-pocket share borne by insured users may in fact be lower even as total spending is higher. They are consistent with moral hazard, the price-responsiveness of dental services demand demonstrated in the RAND Health Insurance Experiment, in which lower cost-sharing increased demand for dental services (29) but may equally reflect an access effect, with coverage enabling needed care, or adverse selection, whereby those anticipating greater dental need are more likely to obtain coverage. Because our analysis is cross-sectional, we cannot disentangle these mechanisms, which the randomized RAND experiment was able to isolate.

Our expenditure estimates should be interpreted in the context of our estimation approach. Direct comparison of our expenditure estimates with those from prior studies is not appropriate, given differences in survey year (and associated price changes), study populations (age range and insurance status), the definition of dental expenditures (payment sources included, and whether estimates are conditional on use or population-wide), and estimation methods. We therefore focus on the consistency of the direction of associations rather than absolute dollar magnitude.

Methodologically, the two-part model is well-suited to dental expenditures because it accommodates the large share of older adults with no spending and the right-skewed distribution among users features that ordinary least squares or log-transformed single-equation models handle less reliably (24, 25)—and it separates the probability of any spending from the level of spending among users while recovering estimates on the original dollar scale. Interpreting the two parts together clarifies how social determinants operate at different stages of care. Lower education and income were associated with both a lower probability of any dental visit and lower expenditures among users, a compounding disadvantage in which socioeconomic barriers limit entry into care and, among those who enter, accompany less intensive treatment.

These findings have policy relevance, though they should be read as associational given the cross-sectional design. Dental insurance coverage emerges as a modifiable determinant of both access and spending, and recent developments point toward expanded oral-health benefits under Medicare. The Centers for Medicare & Medicaid Services has broadened coverage for certain dental services integral to other medical treatments (30), and congressional bills have proposed a fuller Medicare dental benefit (31, 32). At the same time, coverage expansion alone may be insufficient. Medicare's existing dental benefits remain too narrow to meaningfully influence routine or restorative care (33), and even seniors enrolled in Medicare Advantage plans with dental benefits continue to report unmet dental need (11), indicating that benefit adequacy not merely the presence of coverage—shapes access. Embedding oral-health reform within broader Medicare modernization may be necessary to advance equity in aging (34).

Our findings also signal a role for clinicians. Dentists and primary care physicians are positioned to identify older adults facing access barriers and to refer uninsured or underinsured patients to community dental resources, dental school clinics, or charitable care programs. SDOH screening in dental settings has been shown to be feasible and acceptable, although routine adoption by dentists remains limited (35). Addressing oral health inequalities requires embedding oral health within broader health and social-determinant strategies rather than behavioral interventions alone (36).

### Strengths and limitations

A major strength of this study is the use of nationally representative MEPS 2022 data, which provide robust estimates of dental care use and expenditures among U.S. older adults across all race/ethnicities and among those with lower poverty, as MEPS has been oversampling such populations. By using a two-part modeling approach, we captured both the likelihood of having any dental expenditure and the magnitude of spending among users, which better reflects the zero-inflated, skewed nature of cost data (37). The inclusion of multiple SDOH strengthens the analysis by capturing a wide range of structural factors that influence dental care access. Because MEPS is a cross-sectional study, causal inferences cannot be drawn; associations observed between socioeconomic factors, insurance status, and dental spending may reflect unmeasured differences that preceded the survey year. Dental expenditures in MEPS are self-reported and subject to recall bias, which may lead to under- or over-estimation of actual spending. The dataset does not capture clinical dental needs or has not been linked to medical conditions files that capture oral health status, limiting our ability to determine whether lower use reflects lower need or unmet need. Several potential confounders were unmeasured. Most importantly, we lacked clinical oral health and dentate status (e.g., edentulism), which strongly shape both the need for and the type of dental care; edentulous older adults may incur lower restorative spending, potentially confounding SDOH–expenditure associations. We also could not capture dental benefit generosity (annual maximums, cost-sharing) beyond coverage type, local dentist supply, dental anxiety, oral health literacy, or patient preferences. Observed associations may reflect residual confounding or reverse pathways. Because we could not distinguish the mix or complexity of procedures, lower expenditures may reflect lower-cost care (e.g., extractions rather than restorative or prosthodontic treatment) rather than less care overall.

## Conclusion

Nearly one out of two older adults in the United States did not receive any dental care in 2022. Unfavorable SDOH were associated with lower odds of dental care. Favorable SDOH were associated with higher expenditures. Our findings suggest that policies that address more than just expanding dental insurance coverage. Future research with robust and longitudinal study designs is needed to confirm our findings.

## Data Availability

The original contributions presented in the study are included in the article/Supplementary Material, further inquiries can be directed to the corresponding author/s.
