# Supplementary material for: The association of social determinants of health with dental care use and expenditures among older adults in the United States
Source: Front Oral Health. 2026 Oct 29;7:1870576. doi: 10.3389/froh.2026.1870576 (PMC13755213; doi:10.3389/froh.2026.1870576)
Supplement: Supplementary file 1 [file Table1.docx]

Supplementary Material

# 1 Supplementary Table

| **Appendix A. Unadjusted Two-Part Model of Dental Care Use and Expenditures among Older Adults (age ≥ 65 years)**  **Logistic Regressions on Dental Care Use and Generalized Linear Models (GLM) with Gamma Distribution and Log Link**  **Medical Expenditure Panel Survey, 2022** | | | | | | | | | | |
| --- | --- | --- | --- | --- | --- | --- | --- | --- | --- | --- |
|  |  | **Logistic Regression** | | | **GLM** | | | **Marginal Effects** | | |
|  |  | **uOR** | **95% CI** | **p-value)** | **Beta** | **SE** | **P-value** | **ME** | **95% CI** | **P-value** |
| **Sex (Ref = Male)** | | |  |  |  |  |  |  |  |  |
|  | Female | 1.10 | [0.97, 1.25] | 0.151 | -0.11 | 0.13 | 0.408 | -40.76 | [-212.60, 131.08] | 0.641 |
|  | Constant |  |  |  | 7.16 | 0.12 | <0.0001 |  |  |  |
| **Age in Years (Ref = 65-69 years)** | | | |  |  |  |  |  |  |  |
|  | 70-74 years | 1.07 | [0.88, 1.30] | 0.518 | 0.03 | 0.18 | 0.872 | 40.39 | [-215.41, 296.18] | 0.756 |
|  | 75-79 years | 1.08 | [0.86, 1.36] | 0.512 | -0.09 | 0.18 | 0.62 | -35.46 | [-280.41, 209.49] | 0.776 |
|  | 80, Older | 0.71 | [0.58, 0.86] | 0.001 | -0.06 | 0.18 | 0.76 | -137.69 | [-370.57, 95.19] | 0.245 |
|  | Constant |  |  |  | 7.12 | 0.16 | <0.0001 |  |  |  |
| **Race and Ethnicity (Ref = NHW)** | | | |  |  |  |  |  |  |  |
|  | NHB | 0.33 | [0.26, 0.43] | <0.0001 | -0.02 | 0.14 | 0.898 | -338.99 | [-503.93, -174.06] | <0.0001 |
|  | Hispanic | 0.33 | [0.25, 0.45] | <0.0001 | -0.03 | 0.16 | 0.837 | -343.83 | [-514.40, -173.26] | <0.0001 |
|  | Other | 0.50 | [0.35, 0.72] | <0.0001 | -0.31 | 0.16 | 0.05 | -351.59 | [-529.84, -173.34] | <0.0001 |
|  | Constant |  |  |  | 7.12 | 0.07 | <0.0001 |  |  |  |
| **Education (Ref = College)** | | | |  |  |  |  |  |  |  |
|  | LT HS | 0.15 | [0.11, 0.21] | <0.0001 | 0.47 | 0.53 | 0.378 | -340.35 | [-922.65, 241.94] | 0.251 |
|  | HS | 0.29 | [0.24, 0.36] | <0.0001 | -0.09 | 0.12 | 0.444 | -401.04 | [-534.05, -268.02] | <0.0001 |
|  | Some College | 0.54 | [0.43, 0.66] | <0.0001 | -0.08 | 0.09 | 0.373 | -229.68 | [-372.38, -86.98] | 0.002 |
|  | Constant |  |  |  | 7.11 | 0.06 | <0.0001 |  |  |  |
| **Poverty Status (Ref = High Income)** | | | |  |  |  |  |  |  |  |
|  | Poor | 0.27 | [0.21, 0.36] | <0.0001 | -0.14 | 0.17 | 0.414 | -423.82 | [-571.22, -276.42] | <0.0001 |
|  | Low | 0.35 | [0.28, 0.44] | <0.0001 | -0.17 | 0.1 | 0.094 | -376.45 | [-492.57, -260.33] | <0.0001 |
|  | Middle | 0.55 | [0.43, 0.69] | <0.0001 | 0.18 | 0.18 | 0.322 | -57.6 | [-324.90, 209.71] | 0.672 |
|  | Constant |  |  |  | 7.08 | 0.05 | <0.0001 |  |  |  |
| **Dental Insurance (Ref = None)** | | | |  |  |  |  |  |  |  |
|  | Private | 2.04 | [1.70, 2.45] | <0.0001 | -0.03 | 0.13 | 0.79 | 189.15 | [20.63, 357.67] | 0.028 |
|  | Medicare Ony | 1.08 | [0.87, 1.34] | 0.467 | -0.09 | 0.15 | 0.546 | -30.76 | [-215.23, 153.70] | 0.743 |
|  | Constant |  |  |  | 7.13 | 0.11 | <0.0001 |  |  |  |
| **Marital Status (Ref = Married)** | | | |  |  |  |  |  |  |  |
|  | Wid/Div/Sep | 0.62 | [0.53, 0.72] | <0.0001 | 0.23 | 0.15 | 0.127 | -4.89 | [-203.39, 193.60] | 0.961 |
|  | Single | 0.71 | [0.54, 0.93] | 0.014 | -0.03 | 0.13 | 0.818 | -113.43 | [-274.89, 48.03] | 0.168 |
|  | Constant |  |  |  | 7.02 | 0.04 | <0.0001 |  |  |  |
| **Region (Ref = North East)** | | | |  |  |  |  |  |  |  |
|  | Midwest | 1.11 | [0.85, 1.45] | 0.435 | -0.19 | 0.12 | 0.099 | -101.31 | [-288.25, 85.64] | 0.287 |
|  | South | 0.79 | [0.61, 1.02] | 0.072 | -0.16 | 0.18 | 0.392 | -177.7 | [-423.00, 67.59] | 0.155 |
|  | West | 0.91 | [0.70, 1.16] | 0.435 | -0.08 | 0.12 | 0.474 | -91.02 | [-267.67, 85.63] | 0.311 |
|  | Constant |  |  |  | 7.22 | 0.09 | <0.0001 |  |  |  |
| **Multimorbidity (Ref = No)** | | | |  |  |  |  |  |  |  |
|  | Yes | 1.29 | [1.07, 1.54] | 0.006 | 0.03 | 0.13 | 0.819 | 94.18 | [-74.16, 262.52] | 0.272 |
|  | Constant |  |  |  | 7.08 | 0.1 | <0.0001 |  |  |  |
| **Depression (Ref = No)** | | |  |  |  |  |  |  |  |  |
|  | Yes | 0.93 | [0.73, 1.18] | 0.536 | -0.15 | 0.12 | 0.224 | -111.16 | [-258.64, 36.31] | 0.139 |
|  | Constant |  |  |  | 7.12 | 0.07 | <0.0001 |  |  |  |
| **Anxiety (Ref = No)** | | |  |  |  |  |  |  |  |  |
|  | Yes | 1.16 | [0.93, 1.45] | 0.178 | 0.00 | 0.15 | 0.979 | 48.56 | [-156.19, 253.32] | 0.641 |
|  | Constant |  |  |  | 7.10 | 0.07 | <0.0001 |  |  |  |
|  |  |  |  |  |  |  |  |  |  |  |
| **Physical Activity (Ref = Other)** | | | |  |  |  |  |  |  |  |
|  | 5 or more times/week | 0.71 | [0.61, 0.82] | <0.0001 | 0.21 | 0.12 | 0.081 | 33.41 | [-127.88, 194.70] | 0.684 |
|  | Constant |  |  |  | 6.99 | 0.05 | <0.0001 |  |  |  |
| **Tobacco Use (Ref = No)** | | |  |  |  |  |  |  |  |  |
|  | Every Day | 0.30 | [0.21, 0.44] | <0.0001 | 0.17 | 0.33 | 0.611 | -277.22 | [-563.94, 9.50] | 0.058 |
|  | Some days | 0.30 | [0.16, 0.55] | <0.0001 | -0.05 | 0.32 | 0.889 | -354.4 | [-577.02, -131.77] | 0.002 |
|  | Constant |  |  |  | 7.09 | 0.06 | <0.0001 |  |  |  |
| **Polypharmacy (Ref = No Polypharmacy)** | | | |  |  |  |  |  |  |  |
|  | Yes | 1.00 | [0.86, 1.17] | 0.977 | 0.23 | 0.16 | 0.152 | 156.38 | [-88.69, 401.44] | 0.210 |
|  | Constant |  |  |  | 7.03 | 0.05 | <0.0001 |  |  |  |
|  |  |  |  |  |  |  |  |  |  |  |

Note: Based on 5,115 older adults (aged 65 years or older) with health insurance and observed throughout the year in 2022. Missing values in the following variables are not presented: education, tobacco use, and physical activity. Group differences in total dental care and out-of-pocket expenditures were tested using t statistics.

Chron: Chronic conditions; HS: High school; GE: Greater than or equal; mcare: Medicare; Wt: Weighted; Wid/div/sep: Widowed or divorced or separated
